# Facile Tailoring of Structures for Controlled Release of Paracetamol from Sustainable Lignin Derived Platforms

**DOI:** 10.3390/molecules26061593

**Published:** 2021-03-13

**Authors:** Mario Culebras, Mahboubeh Pishnamazi, Gavin M. Walker, Maurice N. Collins

**Affiliations:** 1 Stokes Laboratories, School of Engineering, Bernal Institute and AMBER, University of Limerick, V94 T9PX Limerick, Ireland; mario.culebrasrubio@ul.ie; 2 Pharmaceutical Centre (SSPC), University of Limerick, V94 T9PX Limerick, Ireland; Seyedeh.Pishnamazi@ul.ie (M.P.); Gavin.Walker@ul.ie (G.M.W.)

**Keywords:** lignin, hydrogels, crosslinking, drug release

## Abstract

Nowadays, sustainable materials are receiving significant attention due to the fact that they will be crucial for the development of the next generation of products and devices. In the present work, hydrogels have been successfully synthesized using lignin which is non-valorized biopolymer from the paper industry. Hydrogels were prepared via crosslinking with Poly(ethylene) glycol diglycidyl ether (PEGDGE). Different crosslinker ratios were used to determine their influence on the structural and chemical properties of the resulting hydrogels. It has been found that pore size was reduced by increasing crosslinker amount. The greater crosslinking density increased the swelling capacity of the hydrogels due to the presence of more hydrophilic groups in the hydrogel network. Paracetamol release test showed higher drug diffusion for hydrogels produced with a ratio lignin:PEGDGE 1:1. The obtained results demonstrate that the proposed approach is a promising route to utilize lignocellulose waste for producing porous materials for advanced biomedical applications in the pharmacy industry.

## 1. Introduction

Sustainable development of materials has become on the biggest scientific challenges in the current society [1]. The global warming generated by the increasing CO_2_ emission each year has forced new research lines in order to find renewable sources to produce the next generation of materials that can derive in low environmental impact products and their circular use to avoid waste accumulation in the landfills [1,2]. Looking at this scenario, agricultural and forestry lignocellulose waste, which represents more than 2 billion tons annually [1,3], requires novel routes for their valorization. Cellulose, lignin and hemicellulose are the three major components of lignocellulosic biomass. Cellulose has well stablished path to be valorized in the paper, biomedical and textile industry [4,5,6]. Hemicellulose is being used in the biorefineries to produce biofuels and chemicals [2,7,8]. However, lignin remains underutilized with more than 70 million tons produced each year and only being commercialized 2% [9,10]. Therefore, there has been an increased number of investigations to find new ways to produce valuable products using lignin waste strains. Clear examples are: the use of lignin to synthetize thermosetting resins [11] and carbon fibers for structural composites manufacturing [12,13,14,15]. In addition lignin is currently being use as precursor to produce carbon based nanostructures for energy applications such as: batteries, supercapacitors [16,17] and thermoelectric devices [18]. Lignin is a phenolic polymer consisting of phenyl propanol units that enhances the hydrophobic properties and promotes mineral transport in plants. The structural backbone of the polymer is comprised of three different phenyl-propane monomers, termed sinapyl alcohol, coniferyl alcohol, and ρ-coumaryl alcohol. The ratio of these monomers determines the degree of branching and the reactivity of lignin. The monomers transfer to three phenolic sub-structures, namely syringyl (S), guaiacyl (G), and *p*-hydroxyphenyl (H) units. These phenolic structures contain many functional groups including hydroxyls, carboxyls, carbonyls and methoxyls which can be further chemically modified to tailor lignin properties based on the application. One of chemical modification that can be done in lining structure, is the addition of hydrophilic molecules to produce hydrogels [19]. Hydrogels are three-dimensional network of polymers that can absorb large amounts of water while remain insoluble in aqueous solutions due to chemical or physical crosslinking of individual polymers. Currently, lignin has become the focus of many studies in the field of hydrogel development due to its inherent properties such as biocompatibility, biodegradability, low toxicity, and importantly its susceptibility to enzymatic degradation [20]. For this reason, the formation of hydrogels based on lignin represents an excellent valorization route for lignin waste streams through biomedical and biotechnological applications, including enzyme immobilization, tissue engineering (TE), drug delivery systems, and biosensors, [21,22,23,24,25,26,27,28]. In particular, controlled drug release is an area of interest in pharmaceutical science gaining considerable attention within the research community. Controlled release systems allow tuning of drug dosage to specific rates, this keeps the drug concentration at an effective therapeutic level, thereby maximizing its effect within the body [29,30,31,32].Looking at this scenario, this work has been focused on the development of organosolv lignin derived hydrogels crosslinked with Poly (ethylene glycol) diglycidyl ether (PEGDGE) at different ratios to produce a new platform for drug delivery systems. In addition, structure/property relationships of these hydrogels are investigated to provide complete understanding of lignin-based hydrogels. There is no doubt that this will offer new routes for lignin valorization in the pharmaceutical field minimizing its environmental impact, decreasing CO_2_ emission and contributing to the circular use of resources.

## 2. Results

**Figure 1:**
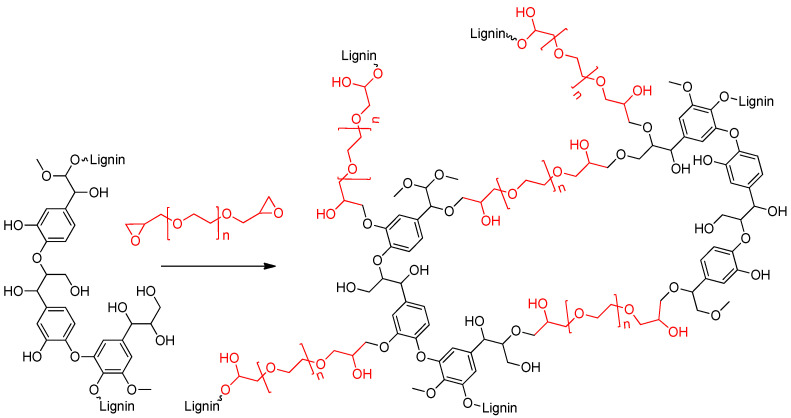
Simplified reaction scheme of crosslinking process between organosolv lignin and PEGDGE.

Organosolv lignin showed excellent solubility in NaOH 3.3M due to the presence of phenolic groups (value) in this particular type of lignin which are deprotonated at basic pH (PKa < 10). Both type of samples crosslinked with 8 and 10 g of PEGDGE (TCA8PEGDGE and TCA10PEGDGE, respectively) gelled after 24 h. The crosslinking process is carried out through the hydroxyl groups of lignin and the propylene oxide groups from PEGDGE as it descried in the schematic of Figure 1.

This reaction derives in a highly interconnected structure with hydrophilic regions due to the presence of multiple ether linkages from the crosslinker PEGDGE. Preliminary studies showed that ratios lignin: PEGDGE lowers than 1:1 did not show good crosslinking behavior producing weak hydrogels as it shown in Figures S1–S4 from the Suplementary Materials.

**Figure 2:**
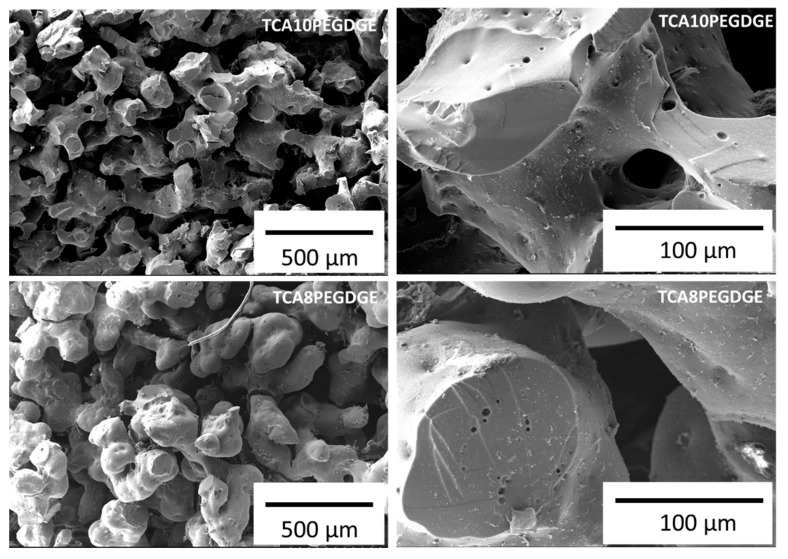
SEM images of freeze-dried lignin based hydrogels showing porous structures at varying PEGDGE levels.

Figure 2 shows the morphology of the freeze-dried hydrogels with two different amounts of PEGDGE used in this study. Both type of hydrogels showed a macro porous structure with interconnected channels. This morphology is typically observed in porous carbon materials derived from lignin [33]. For the case of TCA10PEGDGE samples, the walls of the freeze-dried hydrogels showed a worm-like morphology with an average of particle size around 190 ± 20 µm. similar morphology was observed for case of the hydrogels based on the composition TCA8PEGDGE. However, these samples depicted a larger particle size around 300 ± 15 µm and the walls shower a more rounded morphology.

**Figure 3:**
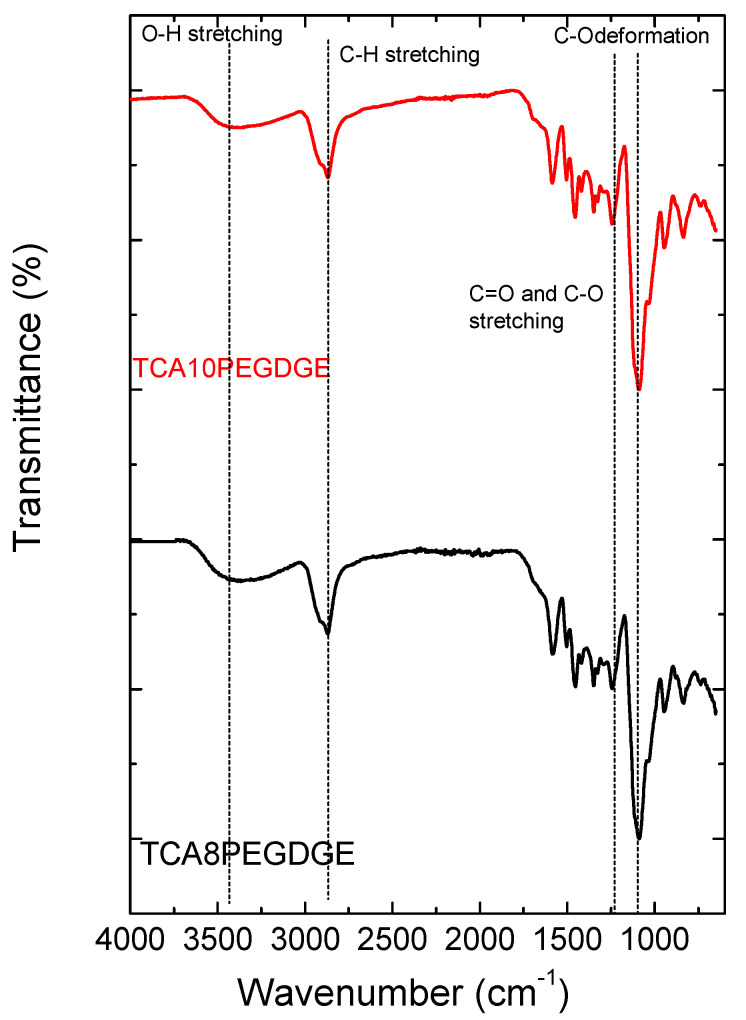
FTIR spectra obtained for the lignin freeze dried hydrogels at two different crosslinker levels.

Figure 3 shows the FTIR spectra of lignin hydrogels in the range from 4000 cm^−1^ to 600 cm^−1^. The typical vibrational modes of lignin derived materials are observed [13,34]. At 3372 cm^−1^ appears the O-H stretching indicating the presence of hydroxyl groups generated when the propylene oxide ring is opened during the crosslinking process. The band centered at 2937 and 2850 cm^−1^ correspond with the symmetrical and asymmetrical C-H stretching of the methyl and methylene groups. Additionally, the C-O deformation in secondary alcohols and aliphatic ethers was observed at bands centered at 1080 cm^−1^ [13,34], being more intense as function of PEGDGE content. This fact evidences the presence of the ether structures coming from the addition of the crosslinker. In addition, the aromatically skeletal vibrations a located at bands centered at 1590–1600 cm^−1^. The presence of C=O and C-O stretching in ether linkage is evidenced at 1200 cm^−1^. 

**Figure 4:**
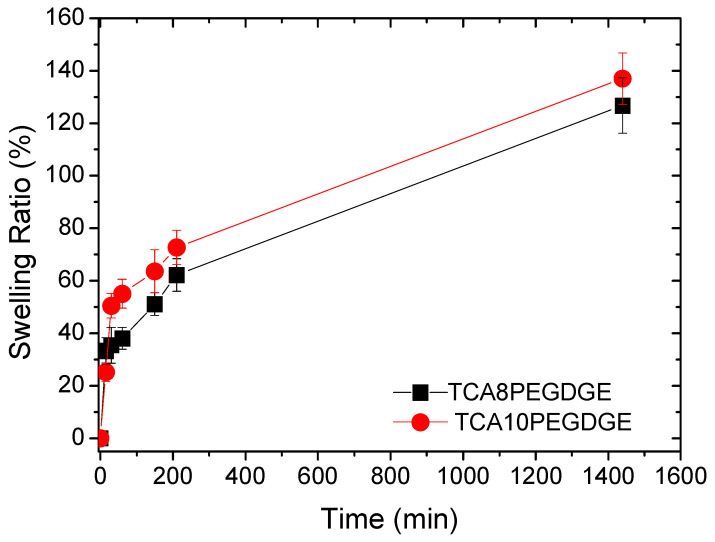
Swelling behavior of lignin based hydrogels at the two optimized crosslinker levels.

The swelling capacity was measured for the different lignin-based hydrogels in PBS. The results are shown in Figure 4. Both hydrogels showed similar swelling capacity indicating that the crosslinking destiny is similar in both hydrogels. Typically increasing crosslinking density reduces swelling capacity, which is related to formation of a network with a greater density and less porosity. This trend has been evidenced in numerous woks published until the date [35,36,37]. However, for this system the swelling mechanism is different since the responsible site for the water intake is the hydrophilic part coming from PEGDGE, due to the hydrophobic nature of lignin at neutral pH (PBS buffer). Therefore, the addition of more crosslinker to the lignin structure increases the number of hydrophilic sites in the reticulated polymer network and in consequence the swilling capacity. This fact can explain the slightly higher values obtained for the sample TCA10PEGDGE. The crosslinking mechanism given by nucleophilic reactions between hydroxyl groups in basic pH with epoxide rings (glycidyl ethers) produces ring-opening reaction generating secondary alcohols groups linked to a long ether chains which plays an important role for water absorption. This support the results obtained in the FTIR analysis that evidence of the presence of hydroxyl groups and the increasing number of ether linkages in the lignin based hydrogels.

**Figure 5:**
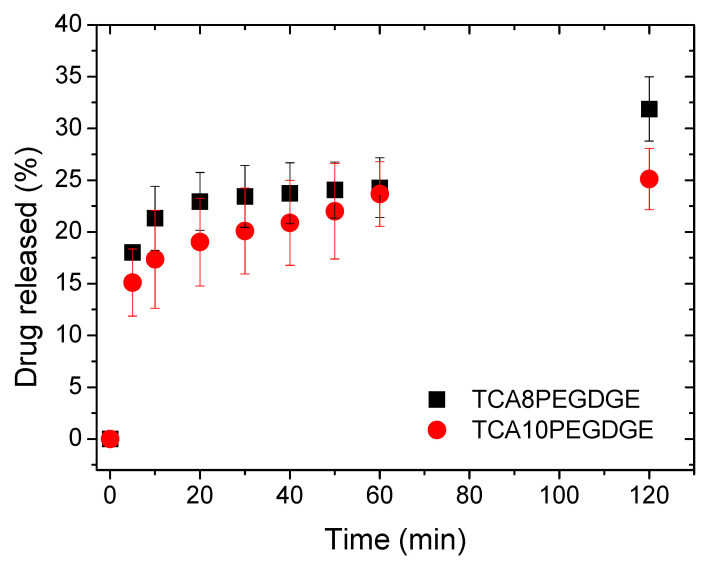
Paracetamol released from each hydrogel at the two crosslinker levels.

Paracetamol was selected as standard drug for the release tests (Figure 5). Platforms for drug release studies were selected as cylindrical shapes due to it very common for differnt at implant sites. The sample TGA10PEGDGE decreased drug release compared to hydrogels produced with 8 g of crosslinker. This fact can be explained due to a higher affinity of paracetamol to TPEGDGE compared to lignin structure. The molecular interactions by hydrogen bonds between the polyether chains and paracetamol molecules are responsible for its slow release. The addition of lower amounts of crosslinker reduces the interaction between paracetamol and the solvated hydrogel network increasing the diffusion of paracetamol from the lignin containing hydrogels to the media. The results showed how the drug release can be controlled through the composition of the hydrogels since the crosslinking process of the hydrogel changed their release behavior. In addition, the release data have been fitted to the Korsmeyer-Peppas model (see Supplementary Materials Figures S5–S6) obtaining a release exponent n < 0.5 indicating a pseudo-Fickian behavior of diffusion [38]. This offers a new opportunity for the valorization of lignin into high value products in particular in the biomedical field as a platform for drug release systems.

## 3. Materials and Methods

### 3.1. Materials

Alcell organosolv hardwood lignin (TCA, Tecnaro GMbH, Ilsfeld, Germany) with a Mw of 4000 g/mol. Sodium hydroxide (NaOH) pellets of greater than or equal to 98% purity was purchased from AppliChem GmbH (Ilsfeld, Germany). Poly (ethylene glycol) diglycidyl ether (Mw 500 g/mol) was purchased from Sigma-Aldrich (St. Louis, MI, United States). Paracetamol (4-acetamidophenol) was purchased from Phion Chemicals.

### 3.2. Synthesis of Lignin based Hydrogels

Lignin (8 g) was initially dissolved in 20 mL of 3.3M NaOH solution. The solution was magnetically stirred for 24 h at 60 °C to dissolve the lignin into the NaOH solution. Then, lignin solutions were loaded with different amount of PEGDGE (8 g and 10 g) as crosslinker. The solutions were magnetically stirred during 15 min for homogeneous mixture. Then, the solutions were poured into 4 cm Petri dishes until completion the crosslinking (24 h). Finally, the crosslinked hydrogels were molded in 1 cm cylinders rinsed several times with deionized water until neutral pH. 

### 3.3. Freeze Draying of Hydrogels

The 2 variants of the hydrogels were freeze dried in a Eurotherm freeze dryer in the conditions described below. Prior to undergoing the freeze-drying process, the hydrogels were stored at −80 °C overnight. The first step was carried out at −30 °C for 8 h at atmospheric pressure followed by the primary Drying at −10 °C for 16 h at 0.1 mBar Finally, the secondary drying was carried out at 20 °C for 2 h at 0.1 mBar.

### 3.4. Characterization

Morphological analysis was carried out by scanning electron microscopy (SEM) in a Hitachi TM-1000 (Hitachi High-Technologies Corporation, Tokyo, Japan). Freeze dried samples were fractured and mounted in the SEM sample holder. Prior to analysis samples were gold sputtered. The accelerating voltage during SEM observation was 15 kV.

Fourier-Transform Infrared Spectroscopy (FTIR) was used for the structural analysis of the samples. FTIR was performed in a PerkinElmer, (Waltham, MA, USA, USA) Spectrum 100 spectrometer with an attenuated total reflectance (ATR) accessory. A total of 4 scans were conducted per test in a range between 4000–650 cm^−1^.

Swelling tests were conducted at 37 °C in a PolyScience (Warrington, PA, USA) water bath in PBS solution. Prior to testing, gels were dried overnight in a Gallenkamp (Kent, United Kingdom) vacuum oven at 600 Bar with the temperature set at 50 °C. Prior to immersing the gels in PBS, their dry weight was recorded using a Sartorius balance. After the gels were placed in the water bath, their weight was measured periodically over the course of 26 h. Before each weighing, the gels were dried on blotting paper to remove any surface water from the gels. The % swelling of the gels was calculated as follows:

(1) $$\begin{array}{c} \%\;Swelling=\frac{W_{s}-W_{d}}{W_{d}}\times 100 \end{array}$$

where *W_s_* is the weight of the sample at each time point and *W_d_* is the dry weight of the sample.

Hydrogels underwent cargo loading in paracetamol solutions with a concentration of 10mg/mL as described in previous works [39]. After drug loading, the gels were placed in baskets in individual chambers of a 900 mL solution in a Pharma Test dissolution machine. The baskets were rotated at a constant speed of 50 rpm. Aliquots of the drug release solutions were measured over time using UV-Vis spectroscopy (Agilent Technologies Cary 60 UV-Vis spectrophotometer, CA, USA). This analysis was performed to determine the paracetamol percentage present in the solution. The duration of each test was 7 h. Data are presented as mean ± standard deviation (s.d.) and analyzed using one-way analysis of variance (ANOVA). *P*-values < 0.05 were considered significant.

## 4. Conclusions

There is doubt that organosolv lignin represent important and abundant raw materials to produce high end wood-derived products. An understanding of their structural, chemical and mechanical behavior is crucial for the development of efficient and robust drug release platforms. Lignin based hydrogels were produced via crosslinking with PEGDGE. Resulting hydrogels display a macro porous structure after freeze drying. The swelling capacity is influenced by the amount of cross linker used in the synthesis, increasing at higher lignin: PEGDGE ratios due to the addition of more hydrophilic groups. Paracetamol was used as model for release studies and release is higher for the sample prepared with a ratio lignin: PEGDGE 1:1 due to lower H-boding interaction with solvated hydrogel network. Overall, these sustainable platforms show promise as next generation sustainable platforms for drug release applications.

## Data Availability

The data presented in this study is available in this article and supplementary material.

## Supplementary Materials

The following are available online at: Figure S1. Storage and loss modules as a function of the time for a lignin hydrogel crosslinked with 4 g of PEGDGE Figure S2. Storage and loss modules as a function of the time for a lignin hydrogel crosslinked with 8 g of PEGDGE. Figure S3. Viscosity as a function of the time for a lignin hydrogel crosslinked with 8 g and 4 g of PEGDGE Figure S4. Pictures of hydrogels in water after 24 h of crosslinking with varying amounts of PEGDGE Figure S5. Fitted data using Korsmeyer–Peppas model for TGA10PEGDGE Figure S6. Fitted data using Korsmeyer–Peppas model for TGA8PEGDGE.
