# Supplementary material for: Facile Tailoring of Structures for Controlled Release of Paracetamol from Sustainable Lignin Derived Platforms
Source: Molecules. 2021 Mar 13;26(6):1593. doi: 10.3390/molecules26061593 (PMC8000009; doi:10.3390/molecules26061593)
Supplement: Supplementary file 1 [file molecules-26-01593-s001.pdf]

Facile Tailoring of Structures for Controlled Release of Parace-tamol from Sustainable Lignin Derived Platforms

Mario Culebras [1], Mahboubeh Pishnamazi [2] Gavin M. Walker[2] and Maurice N. Collins [1,*]

[1] Stokes Laboratories, School of Engineering, Bernal Institute, University of Limerick, V94 T9PX, Ireland.

[2] Pharmaceutical Centre (SSPC), University of Limerick, Limerick, V94 T9PX, Ireland

* Correspondence: Maurice.collins@ul.ie;

Rheological analysis

The rheological analysis was carried a hybrid rheometer (TA Instruments, USA). The samples were loaded with different crosslinker amounts between disposable 25 mm steel rheological plates with a measurement gap of 1000 µm. The samples were tested in a time sweep mode the strain at a constant value of 2%.

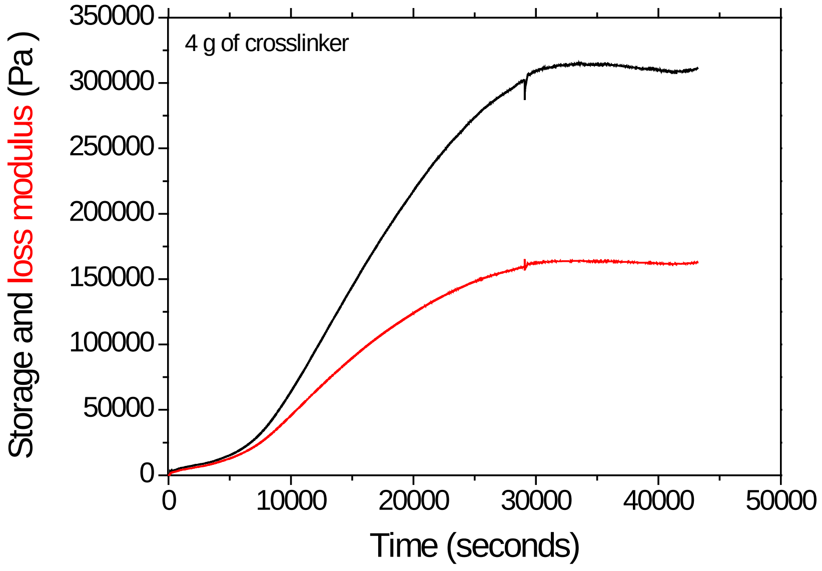

Figure S1. Storage and loss modules as a function of the time for a lignin hydrogel crosslinked with 4 g of PEGDGE.

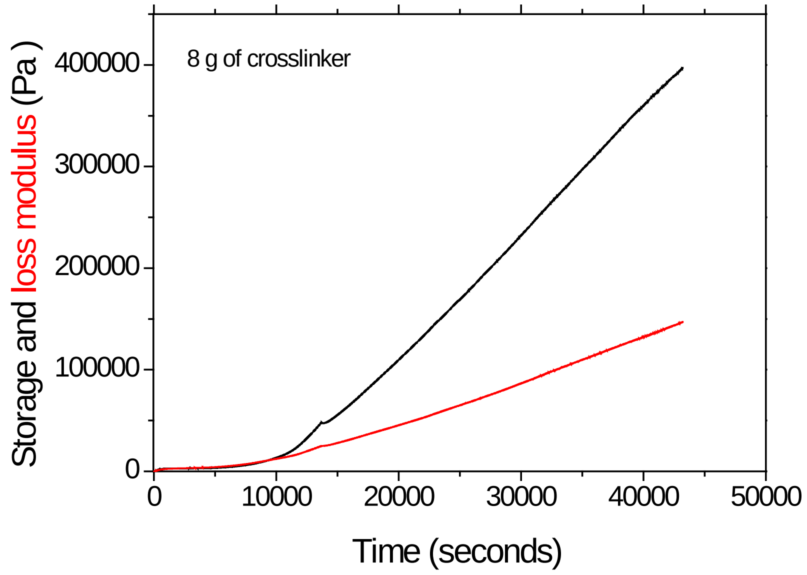

Figure S2. Storage and loss modules as a function of the time for a lignin hydrogel crosslinked with 8 g of PEGDGE.

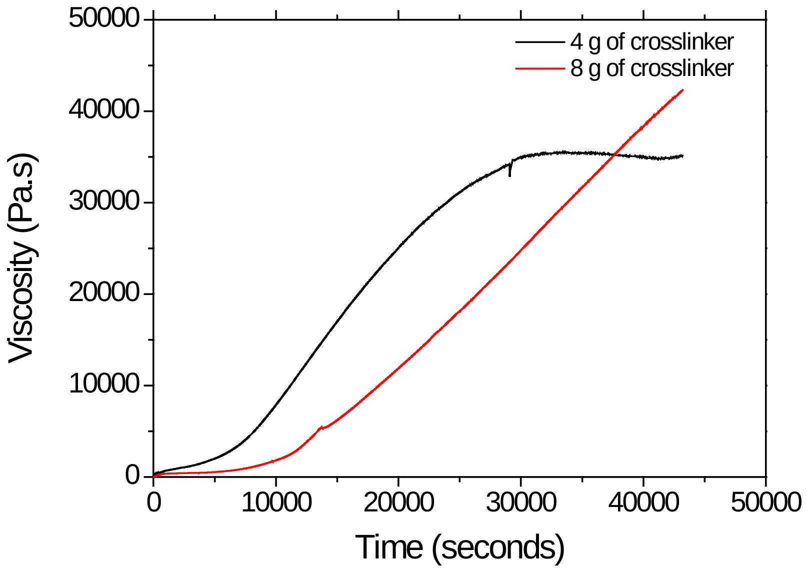

Figure S3. Viscosity as a function of the time for a lignin hydrogel crosslinked with 8 and 4 g of PEGDGE.

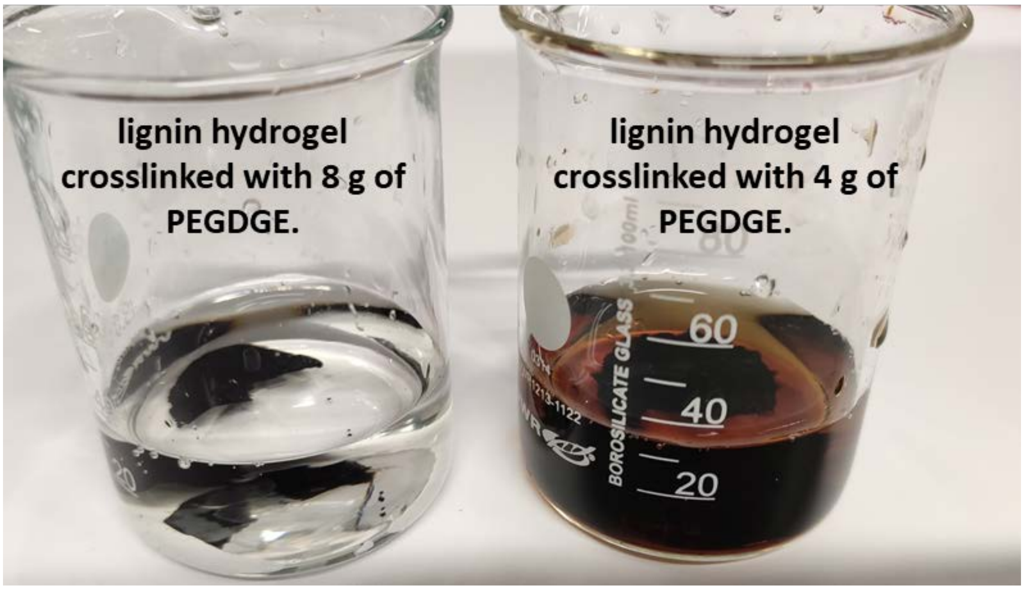

Figure S4. Pictures of hydrogels in water after 24 hours of crosslinking with different amounts of PEGDGE.

Fitting the release data using Korsmeyer–Peppas model

The Korsmeyer–Peppas model is defined by the following equation:

$$\frac{Mt}{M\infty} = kt^n \quad (1)$$

Were where Mt and M∞ are the mass of solute released at time t and the initial mass of solute loaded in the nanofibers, respectively. k is kinetic constant which is related to the properties of delivery system and the encapsulated substance. n is release exponent which is related to the type of transport, geometry and polydispersity of solute, and illustrates the transport mechanism of solute.

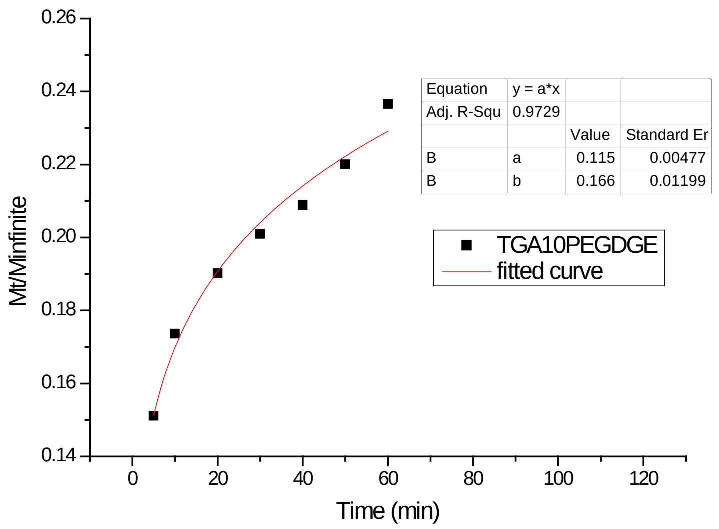

Figure S5. Fitted data using Korsmeyer–Peppas model for TGA10PEGDGE.

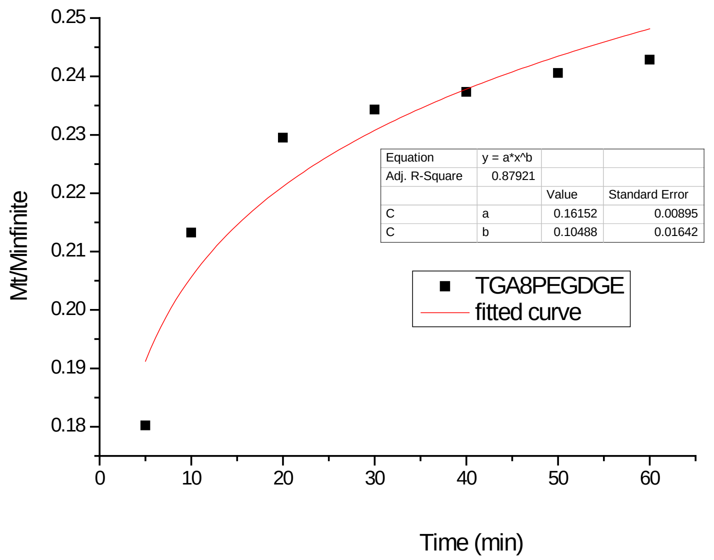

Figure S6. Fitted data using Korsmeyer–Peppas model for TGA8PEGDGE.
